# High-dose rifampicin kills persisters, shortens treatment duration, and reduces relapse rate *in vitro* and *in vivo*

**DOI:** 10.3389/fmicb.2015.00641

**Published:** 2015-06-23

**Authors:** Yanmin Hu, Alexander Liu, Fatima Ortega-Muro, Laura Alameda-Martin, Denis Mitchison, Anthony Coates

**Affiliations:** ^1^Institute for Infection and Immunity, St George’s, University of LondonLondon, UK; ^2^Centre for Clinical Magnetic Resonance Research, University of OxfordOxford, UK; ^3^GlaxoSmithKline Research and Development, Diseases of Developing WorldMadrid, Spain

**Keywords:** *Mycobacterium tuberculosis*, rifampicin, persistence, resuscitation promoting factors, mouse model

## Abstract

Although high-dose rifampicin holds promise for improving tuberculosis control by potentially shortening treatment duration, these effects attributed to eradication of persistent bacteria are unclear. The presence of persistent *Mycobacterium tuberculosis* was examined using resuscitation promoting factors (RPFs) in both *in vitro* hypoxia and *in vivo* murine tuberculosis models before and after treatment with incremental doses of rifampicin. Pharmacokinetic parameters and dose-dependent profile of rifampicin in the murine model were determined. The Cornell mouse model was used to test efficacy of high-dose rifampicin in combination with isoniazid and pyrazinamide and to measure relapse rate. There were large numbers of RPF-dependent persisters *in vitro* and *in vivo*. Stationary phase cultures were tolerant to rifampicin while higher concentrations of rifampicin eradicated plate count positive but not RPF-dependent persistent bacteria. In murine infection model, incremental doses of rifampicin exhibited a dose-dependent eradication of RPF-dependent persisters. Increasing the dose of rifampicin significantly reduced the risk of antibiotic resistance emergence. In Cornell model, mice treated with high-dose rifampicin regimen resulted in faster visceral clearance; organs were *M. tuberculosis* free 8 weeks post-treatment compared to 14 weeks with standard-dose rifampicin regimen. Organ sterility, plate count and RPF-dependent persister negative, was achieved. There was no disease relapse compared to the standard dose regimen (87.5%). High-dose rifampicin therapy results in eradication of RPF-dependent persisters, allowing shorter treatment duration without disease relapse. Optimizing rifampicin to its maximal efficacy with acceptable side-effect profiles will provide valuable information in human studies and can potentially improve current tuberculosis chemotherapy.

## Introduction

Tuberculosis remains a major cause of mortality worldwide (World Health Organization [WHO], 2010). Effective disease control is hindered by bacterial persistence, which necessitates prolonged multi-agent antimicrobial therapy; leading to poor patient compliance, high relapse rates and drug-resistance (Mitchison, 2005). Thus, shortening the duration of chemotherapy is of significant clinical benefit. Drug discovery, although exciting, is arduous, expensive, and time-consuming (Nunn et al., 2008; van Niekerk and Ginsberg, 2009). Therefore, increasing the therapeutic dose of currently marketed antibiotics represents an innovative concept. Of the current anti-tuberculous drugs, only rifampicin is effective against persistent *Mycobacterium tuberculosis* (Mitchison, 1985; Hu et al., 2000; Mitchison, 2000; Mitchison and Fourie, 2010) and has favorable toxicity profile to be used at higher doses (Girling, 1978; Yew and Leung, 2006). Previous studies showed that high-dose rifampicin therapy up to 35 mg/kg is well-tolerated in man (Kreis et al., 1976; Diacon et al., 2007; Boeree et al., 2013) and increases the rate of tuberculosis clearance (Kreis et al., 1976). Similar observations were made in mice (Jayaram et al., 2003; Rosenthal et al., 2012; de Steenwinkel et al., 2013), with a maximum tolerable dose of 160 mg/kg per day (de Steenwinkel et al., 2013). However, whether these effects are attributed to eradication of persistent bacteria, the fundamental problem preventing effective disease control, is unclear.

In this study, we hypothesed that high-dose rifampicin in the current tuberculosis therapy would be [1] effective in eradicating persistent *M. tuberculosis* and [2] prevent emergence of drug resistance and disease relapse. To this end, we rigorously studied the therapeutic effects of incremental doses of rifampicin singly or in combination with isoniazid and pyrazinamide on both *in intro* and *in vivo* models of bacterial persistence.

## Materials and Methods

### *In Vitro* Hypoxia Model

*M. tuberculosis* strain H37Rv was grown in 7H9 medium containing 0.05% Tween 80 supplemented with 10% albumin dextrose complex (ADC; Becton and Dickinson, UK) at 37°C without disturbance for 200 days (Hu et al., 2000). At different time point, CFU counts were performed by plating a serial of 10-folds dilutions of the cultures on 7H11 agar medium supplemented with oleic albumin dextrose complex (OADC, Becton and Dickinson, UK). The 7H11 agar plates were made according to the manufacturer’s instruction. The quality control of the plates for each batch was made by plating a serial 10-folds dilution of a log phase *M. tuberculosis* culture on the agar plates and compared with the 7H11 agar plates from Becton and Dickinson, UK. Colonies were counted after incubation of the plates at 37°C for 3–4 weeks and viability was expressed as Log CFU/ml. The susceptibility patterns of the strain to anti-TB drugs defined as minimum inhibitory concentration (MIC) are listed as following: rifampicin 0.5 mg/L, rifabutin 0.0625 mg/L, rifapentine 0.25 mg/L, isoniazid 0.25 mg/L, ethambutol 2 mg/L, streptomycin 2 mg/L, para-aminosalicylic acid 0.5 mg/L and pyrazinamide > 512 mg/L (pH 5.5–5.6).

### Antibiotic Exposure *In Vitro*

Rifampicin (Sanofi Aventis) at different concentrations was added into log-phase and stationary-phase cultures in the hypoxia model and incubated at 37°C. At different time point, the cultures were washed with phosphate buffered saline (PBS) for three times and viability was determined using CFU counts or broth counts.

### Resuscitation of *M. Tuberculosis*

For resuscitation of *M. tuberculosis* grown *in vitro* and *in vivo*, culture supernatant containing resuscitation promoting factors (RPFs) or 7H9 medium was used as described previously (Mukamolova et al., 2010). *M. tuberculosis* H37Rv was grown in 7H9 medium for 15–20 days until an optical density of 1 was reached. The cultures were harvested by centrifugation at 3000 *g* for 15 min and filtered with 0.2 μm filter (Sartorius) twice. The sterilized culture filtrates were used immediately for the broth counting of the most probable number (MPN) of the bacilli. Broth counting was performed as serial 10-folds dilutions in triplicate in which 0.5 ml of *in vitro* cultures were added to 4.5 ml of the culture filtrates or 0.3 ml of tissue homogenates to 2.7 ml of the culture filtrates. At 10-days intervals over a 2-months period of incubation at 37°C, the broth cultures were examined for visible turbidity. Growth of *M. tuberculosis* in turbid tubes was confirmed by colonial morphology on 7H11 agar plates. The MPN of viable bacilli was then estimated from the patterns of positive and negative tubes (Mukamolova et al., 2010). The absence of microorganisms other than mycobacteria from turbid tubes was shown by plating on blood agar medium (Oxoid) and sabouraud dextrose agar (Oxoid).

### Pharmacokinetic of Rifampicin in BALB/S Mice

Pharmacokinetic (PK) of rifampicin in non-infected mice was determined by dosing-ranging studies. Rifampicin at 0, 10, 20, 30, 40, and 50 mg/kg body weight was administered orally with a single dose by gavage to three BALB/c mice (6–8 weeks old, female) which were obtained from Harlan UK Ltd. Serial venous blood samples (20 μl) were collected at various time points from 1, 2, 3, 4, 5, 6, 7, 8 and 24 h post-treatment by tail puncture and mixed with 40 μl of water. The blood samples were stored at -80°C and subsequently transported in dry ice to GlaxoSmithKline for bioanalysis and PK calculations. Three animals were used for the entire blood sampling process. The concentration of rifampicin in the blood was determined by UPLC-MSMS assay. PK analysis was performed with Phoenix WinNonLin software (version 6.3; Pharsight, USA). The PK parameters were calculated using a non-compartmental analysis (NCA) model.

### Rifampicin Monotherapy in Mouse Model

Rifampicin was tested with five different doses singly for 12 weeks in BALB/c mice (6–8 weeks old, female). The mice were infected intravenously with 1.2 × 10^5^ CFU of the *M. tuberculosis* H37Rv which was mouse-passaged to retain the virulence of the strain. Spleens and lungs from four mice were removed rapidly after sacrifice and sterile autopsy performed at timepoint zero. CFU counts of the organs were performed from serial 10-folds dilutions of the homogenate on plates of selective 7H11 medium and incubated at 37°C for 3–4 weeks. At 2 weeks after infection, the mice were treated with 10, 15, 20, 30, and 50 mg/kg of rifampicin 5 days/week for 12 weeks. At 2nd, 4th, 6th, 8th, 10th, and 12th weeks post-treatment, organ CFU counts from four mice from each treatment group were determined. All animal experiments were performed according to the Animals Scientific Procedures Act, 1986 (an Act of the Parliament of the United Kingdom 1986 c. 14; Home Office Project licence Number 70/7077) with approval from St George’s, University of London ethics committee. The animal husbandry guidelines and animal procedure for this study were followed according to the Animals Scientific Procedures Act, 1986.

### Selection of Rifampicin-Resistant Mutants *In Vivo*

At 8th, 10th, and 12th weeks post-treatment, the lung and spleen homogenates were plated on 7H11 plates containing rifampicin concentration at a serial of twofolds dilution from 0 to 64 mg/L. Colonies from the plates containing MIC value higher than fourfolds were picked and regrown in 7H9 medium. MIC was retested on rifampicin containing 7H11 agar plates.

### Cornell Model

For the combination of rifampicin with isoniazid and pyrazinamide, the Cornell mouse model was used as described previously (McCune and Tompsett, 1956; McCune et al., 1966; Hu et al., 2000). Briefly, at 3 weeks after *M. tuberculosis* H37Rv infection, treatment was given to female BALB/c mice for 14 weeks with 150 mg/kg pyrazinamide, 25 mg/kg isoniazid combined with 10 or 50 mg/kg rifampicin by daily oral administration for 5 days per week. A sample of four mice was sacrificed at the 2nd, 4th, and 6th weeks and a sample of 10 mice was sacrificed 8th, 11th, and 14th weeks of treatment to monitor CFU counts. The organ homogenates from the 14th weeks were cultured in selective Kirchner liquid medium by the addition of polymyxin B 200 U/ml, carbenicillin 100 mg/L, trimethoprim 20 mg/L, and amphotericin B 10 mg/L (Selectatab, Mast Diagnostica GmbH) for 4 weeks with subsequent sub-culturing onto selective Löwenstein-Jensen slopes for a further 4 weeks. Immediately after termination of 14 weeks of chemotherapy, the remaining mice were administered 0.5 mg/mouse of hydrocortisone acetate (McCune et al., 1966) by daily oral administration for 8 weeks to inhibit the host immune response, CFU counts from lungs and spleens were performed from serial 10-folds dilutions of the homogenate and the entire homogenates of the organs were also plated on 7H11 agar plates to determine disease relapse.

### Statistical Analysis

The difference between different experimental groups was determined by analysis of variance (ANOVA) and Student’s *t*-test. The difference between the relapse rates was determined by Chi-square test. *P*-values < 0.05 were considered significant.

## Results

### RPF-Supernatant Resuscitates Persistent *M. Tuberculosis* Growth *In Vitro*

**FIGURE 1:**
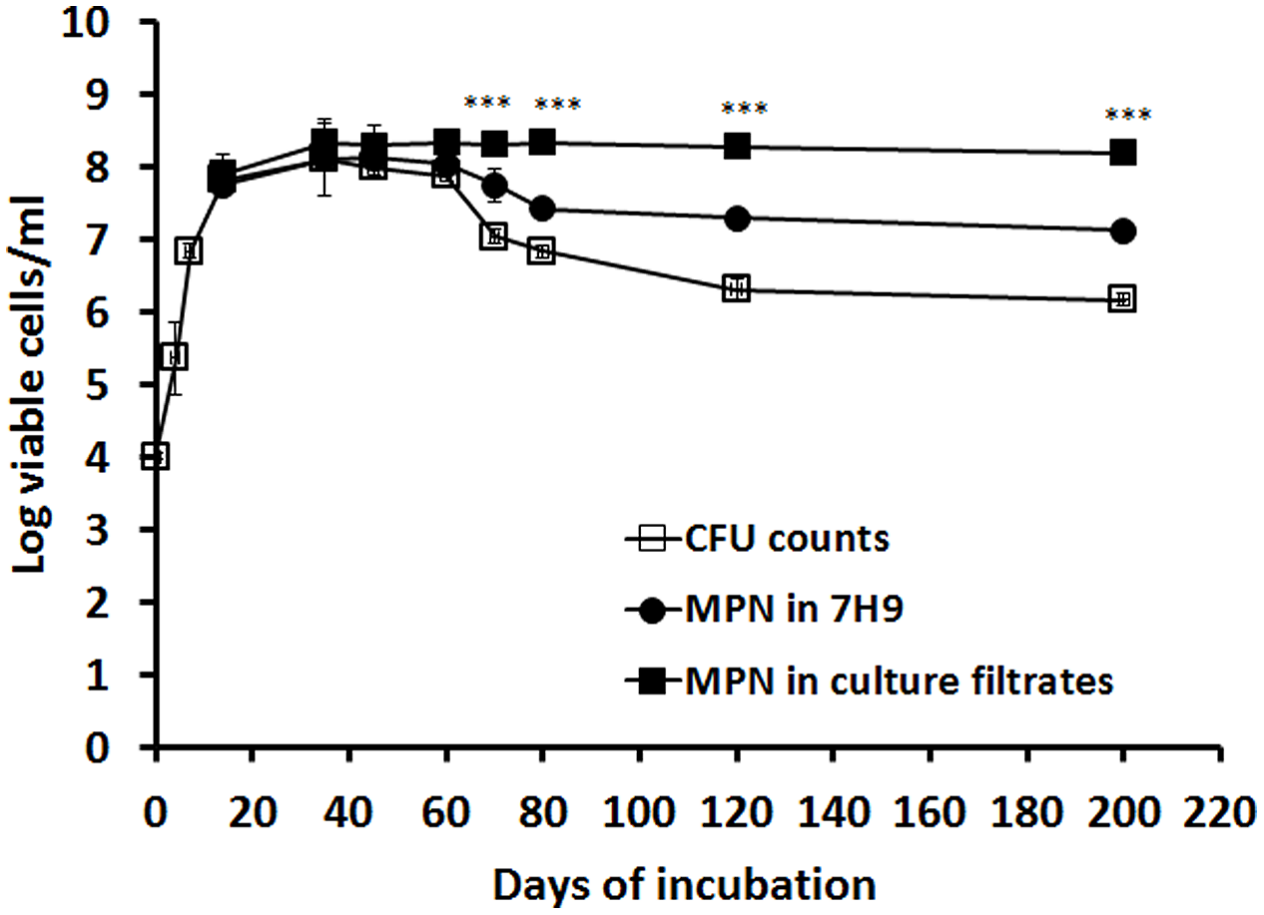
**Resuscitation of RPF-dependent tubercle bacilli from the *in vitro* hypoxic model of *M. tuberculosis*.** *M. tuberculosis* H37Rv was grown in 7H9 medium without disturbance for 200 days. CFU counts were performed at different time points in triplicate (line with empty squares). MPN counts from cultures of 14, 35, 45, 60, 70, 80, 120, and 200 days were performed with 7H9 medium (line with solid circles) or the culture filtrate (line with solid squares). These experiments were performed three times with reproducible results. ANOVA analysis demonstrated that there were significant differences amongst CFU counts, 7H9 broth counts and RPF broth counts (^∗∗∗^*P* < 0.001 to 0.0001).

In the *in vitro* hypoxic model (Wayne, 1976), exponential growth was observed up to 15 days, followed by growth stabilization until day 60, when a 2-log reduction of CFU counts occurred lasting up to day 200 (**Figure 1**). To determine the cause of this count reduction (cell death or viable but non-culturable state), log-phase and stationary phase culture were incubated with the supernatant of a late log-phase culture containing RPF or 7H9 (Mukamolova et al., 2010). These RPF-supernatant or 7H9 medium resuscitated cultures were quantified using the MPN method. Interestingly, at the time points selected for comparison (14, 35, 45, 60, 70, 80, 120, and 200 days), RPF-supernatant resuscitated cultures displayed progressively higher broth counts than the CFU counts (0.08, 0.23, 0. 30, 0.45, 1.28, 1.51, 1.99, and 2.03 logs, respectively; **Figure 1**) compared to those of cultures resuscitated by 7H9 (-0.08, 0.00, 0.13, 0.15, 0.72, 0.82, 0.99 and 0.96 logs, respectively; **Figure 1**). The 7H9 or RPF-dependent persisters were unable to form colonies during prolonged incubation periods. Significant differences were observed amongst CFU counts, 7H9 broth counts and RPF broth counts (*P* < 0.001 to 0.0001) determined by ANOVA analysis.

### Higher Concentration of Rifampicin was Required to Treat, but not Eradicate, *M. Tuberculosis* in Stationary Phase *In Vitro*

**FIGURE 2:**
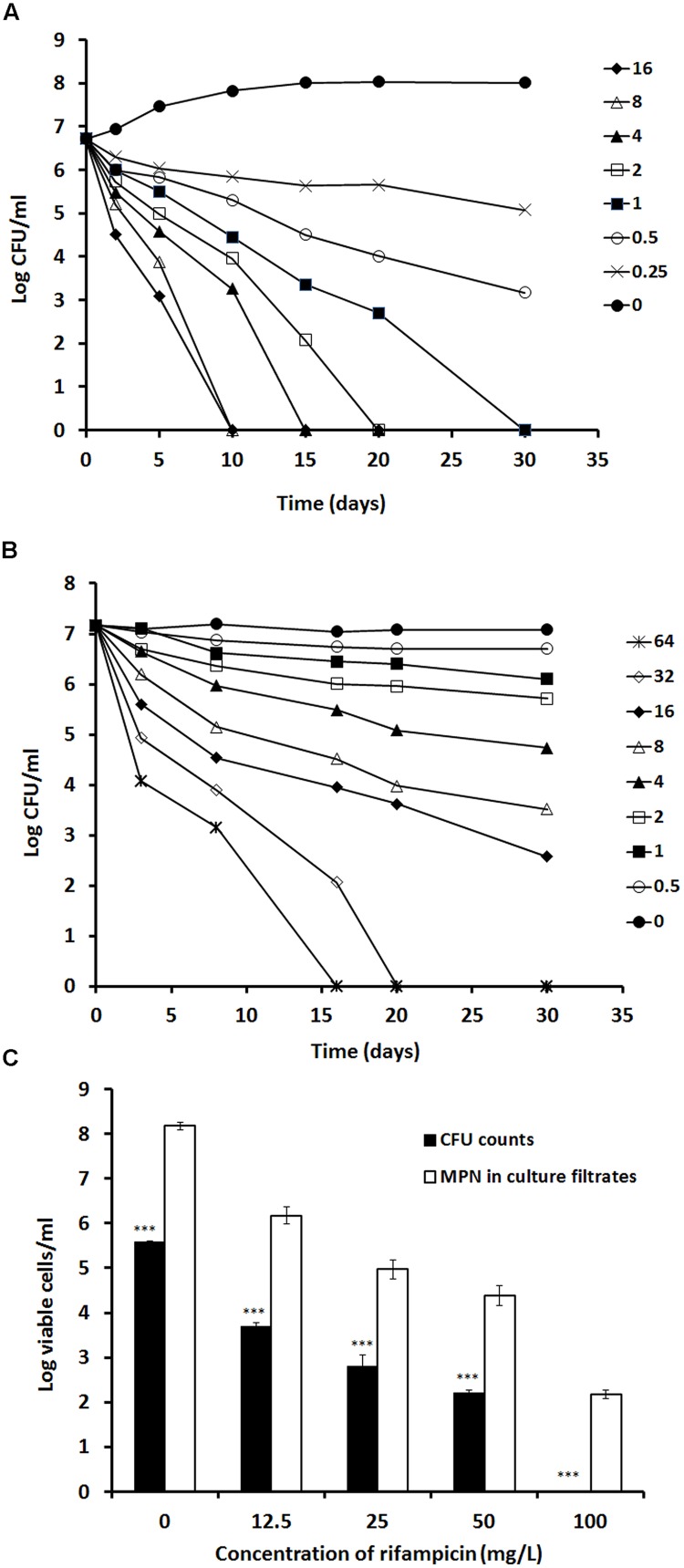
**Determination of rifampicin activity against log-phase and stationary-phase *M. tuberculosis* using time kill curve analysis.** Rifampicin at the concentration from 16 to 0 mg/L were incubated with a 7 days log-phase culture **(A)** and rifampicin at the concentration from 64 to 0 mg/L were incubated with the 60 days stationary-phase culture **(B)**. CFU counts were estimated at different time points. A 100-days culture was treated with 12.5, 25, 50, and 100 mg/L of rifampicin for 5 days. After removal of rifampicin, the treated culture were subject to CFU counting on agar plates and MPN counting with the culture filtrates **(C)**. These experiments were performed three times with reproducible results. Statistical analysis demonstrated that the decline of CFU counts after treatment with different concentrations of rifampicin was significant, *P* < 0.0008 (ANOVA), for the log-phase culture **(A)**, *P* < 0.001 (ANOVA) for the stationary phase culture **(B)** and *P* < 0.001 (^∗∗∗^ Student’s *t*-test) for the 100-days culture **(C)**.

To determine the optimal concentration of rifampicin required to clear stationary-phase bacteria, we examined the effect of incremental drug concentrations on both log- (7 days) and stationary-phase (60 days) cultures; see **Figure 2**. In log-phase cultures, *apparent* bacterial clearance was achieved at/before 20 days with rifampicin concentrations of >2 mg/L (**Figure 2A**). However, to achieve the same antimicrobial effect in stationary-phase cultures, significantly higher drug concentrations (>32 mg/L) were required (**Figure 2B**).

To delineate the level of residual viable *M. tuberculosis* remaining after *apparent* bacterial clearance with high concentration rifampicin treatment, we incubated 100-days cultures with incremental concentrations of rifampicin (12.5, 25, 50, and 100 mg/L) for 5 days. After resuscitation with RPF-culture filtrate, viable bacteria were salvageable from all cultures and increased with reducing concentrations of rifampicin treatment (**Figure 2C**). Importantly, the *apparent* bacterial clearance produced by a 5-days treatment with 100 mg/L rifampicin showed a 2-log resuscitatable growth count with RPF-culture filtrate (**Figure 2C**). This reflects the difficulties in completely eradicating *M. tuberculosis*, even with extremely high rifampicin concentrations, *in vitro*.

### RPF-Supernatant Resuscitates Persistent *M. Tuberculosis* Growth *In Vivo*

**FIGURE 3:**
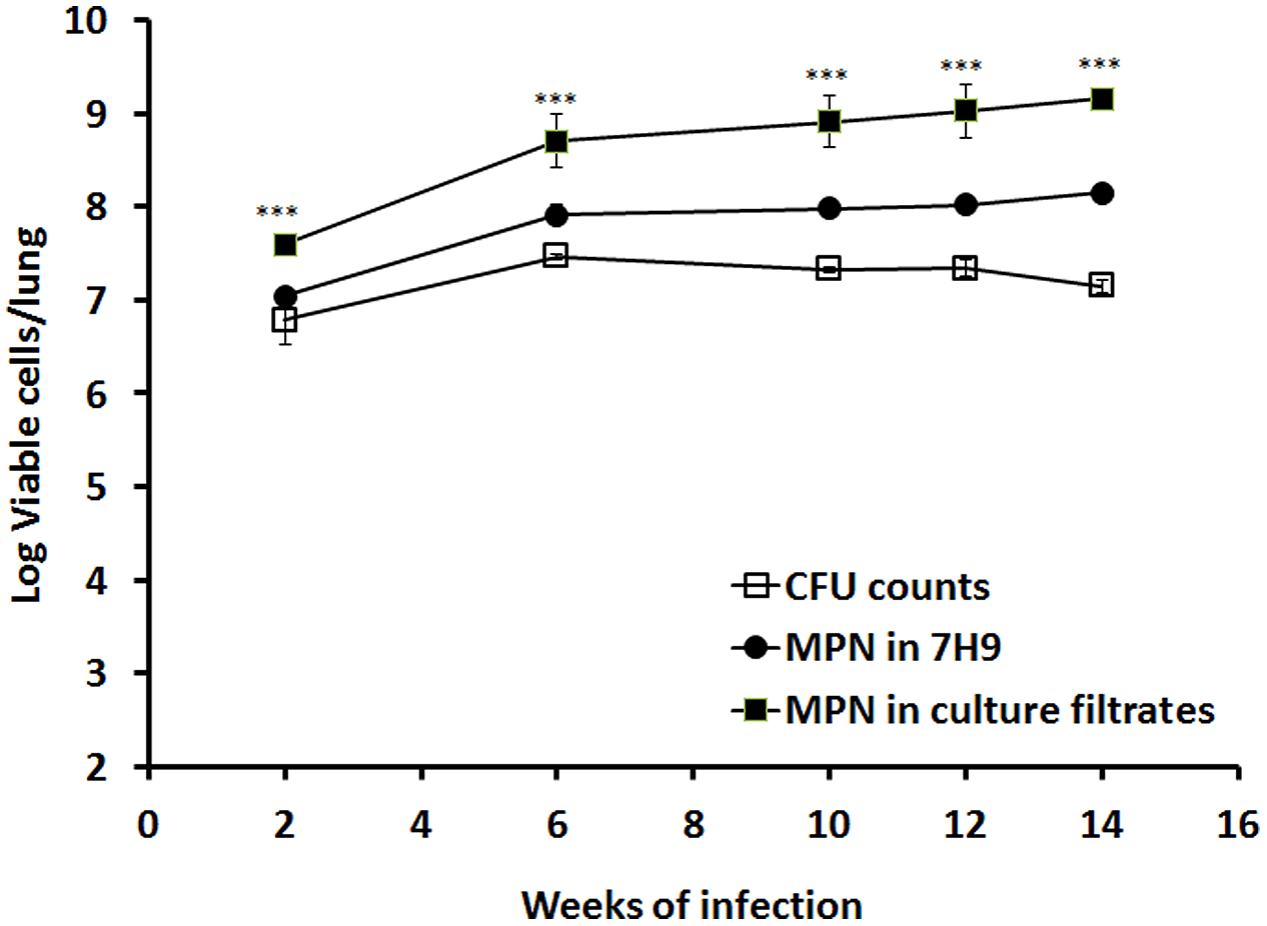
**Resuscitation of *M. tuberculosis* grown in mouse lungs.** BALB/c mice were infected with *M. tuberculosis*. Viability of the bacilli in lung was determined by CFU counting and MPN counting with 7H9 or the culture filtrates at 2, 6, 10, 12, and 14 weeks post-infection. The results have been performed twice with reproducible results. ANOVA analysis demonstrated that there were significant differences amongst CFU counts, 7H9 broth counts and RPF broth counts (^∗∗∗^*P* < 0.001) in mice.

We previously showed that *M. tuberculosis* enters stationary-phase growth after ∼2–3 weeks of infection in mice (Hu et al., 2006). In order to investigate the growth behavior of stationary-phase bacteria *in vivo*, we incubated murine lung homogenates from 2, 6, 11, 12, and 14 weeks of *M. tuberculosis* infection with 7H9 medium or RPF-culture filtrate and determined the bacilli MPN. As illustrated in **Figure 3**, 7H9 medium resuscitated cultures showed higher broth counts than the CFU counts from mouse lungs (0.26, 0.45, 0.65, 0.68, and 1.00 logs, respectively). In contrast, RPF-supernatant resuscitated cultures displayed progressively higher broth counts than the CFU counts from the organ (0.81, 1.24, 1.58, 1.69, and 2.00 logs, respectively). This is the first *selective* demonstration of viable RPF-dependent bacilli in murine tuberculosis infection. There were significant differences amongst CFU counts, 7H9 broth counts and RPF broth counts (*P* < 0.001) in mice determined by ANOVA analysis.

### Pharmacokinetic of Rifampicin

Rifampicin plasma concentration time curves examined over a period of 24 h in BALB/c mice (**Table 1**) indicated a dose-proportional increase in maximal concentration of rifampicin (Cmax) and drug exposure (AUC; **Table 1**). This implies the free (protein unbound) fraction of rifampicin in plasma. Interestingly, Tmax was shortened with the increased dosage of the drug.

**Table 1:** Blood pharmacokinetics parameters of rifampicin in BALB/c mice.

Dose (mg/kg)	Cmax (mg/L)	Tmax (h)	AUC_0-24__h_ (mg^∗^h/L)	AUC_inf_ (mg^∗^h/L)
10	11 ± 2	2.7	121 ± 10	162 ± 29
20	21 ± 1	2.3	228 ± 28	259 ± 36
30	32 ± 2	2	332 ± 31	370 ± 43
40	48 ± 4	1.7	530 ± 33	598 ± 58
50	52 ± 10	1	600 ± 81	739 ± 72

Cmax, maximum blood concentration; Tmax, time to maximum concentration; AUC_*0*-*24*__*h*_, area under the curve from rifampicin administration to 24 h; AUC_*inf*_, area under the curve from rifampicin administration to the infinite time. The values are expressed, except for Tmax, as mean (standard deviation) of the values derived from three mice.

### Effect of High-Dose Rifampicin *In Vivo*

**FIGURE 4:**
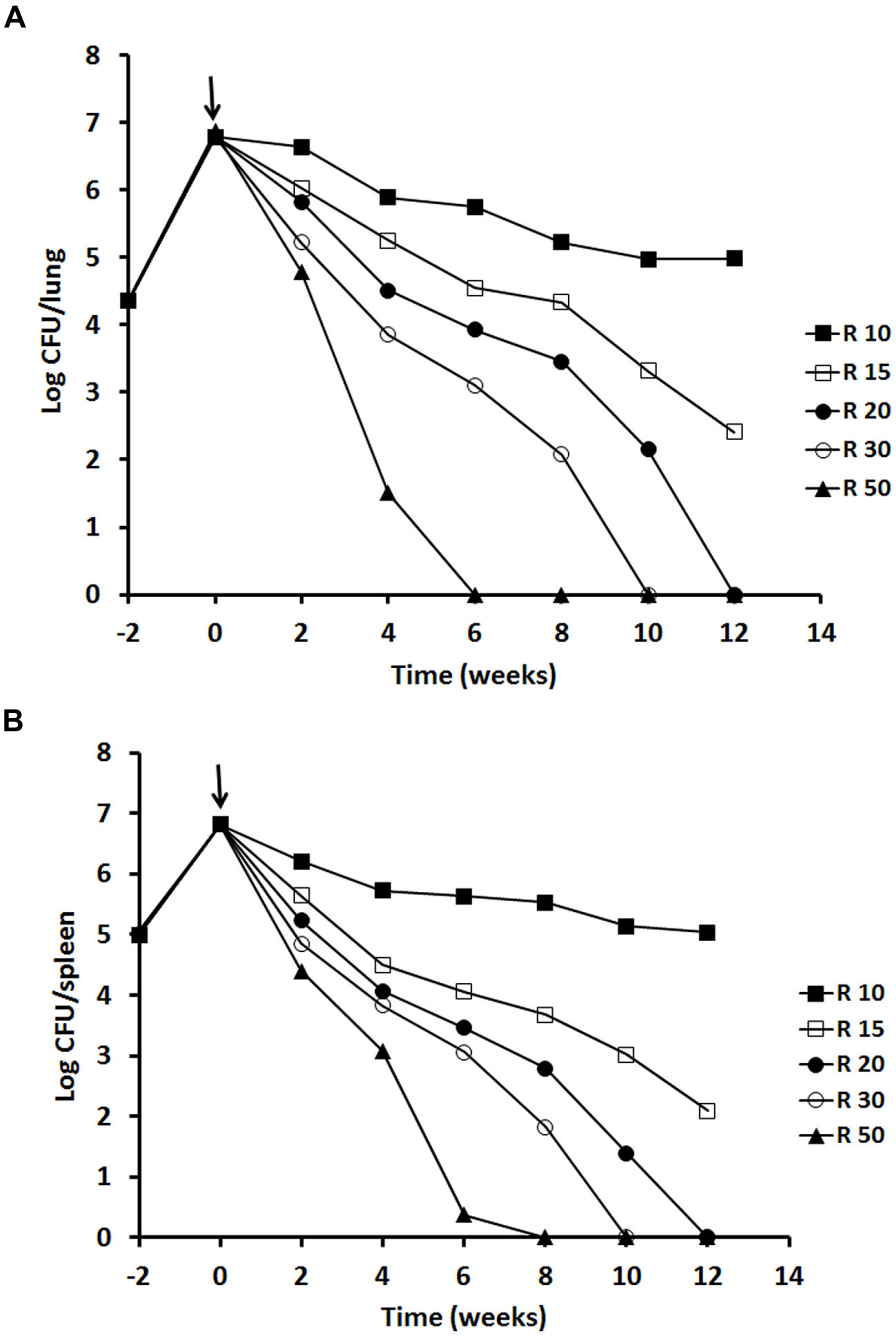
**Viability of *M. tuberculosis* H37Rv in BALB/c mice after rifampicin treatment.** The results of a single experiment are shown with viability expressed as log CFU counts per lung **(A)** and spleen **(B)**. Mice were infected intravenously and the infection was allowed to progress for 2 weeks prior to treatment with rifampicin at 10, 15, 20, 30 and 50 mg/kg indicated as a solid arrow for 12 weeks (time weeks 0–12). At week 2, 4, 6, 8, and 12 of post-treatment, four mice from each group were sacrificed for CFU counting. Data of the mice containing rifampicin-resistant strains for the 10 mg/kg group was excluded. ANOVA analysis demonstrated that the decline of CFU counts after treatment with different concentrations of rifampicin was significant in lungs *P* < 0.02 and in spleens *P* < 0.04.

We investigated the effect of rifampicin dose increments on the rate of bacterial eradication and emergence of drug resistance. At 10 mg/kg, the rate of pulmonary bacterial eradication was slow (99% kill at 8 weeks followed by a plateau). Treatment with 15 mg/kg doubled the rate of bacterial eradication (99% kill at 4 weeks). Both doses failed to achieve undetectable *M. tuberculosis* CFU counts in murine lungs after 12 weeks treatment. Undetectable CFU counts in lungs were achieved with 20, 30, and 50 mg/kg of rifampicin treatment for 12, 10, and 6 weeks, respectively (**Figure 4A**). A similar dose response trend was observed in spleen (**Figure 4B**). No outward signs of toxicity or abnormal behavior were observed in any mice treated with rifampicin.

No rifampicin-resistant strains were detected for the 15, 20, 30, and 50 mg/kg treatment groups. The isolates were susceptible to rifampicin with a MIC at 0.5 mg/L. However, for the 10 mg/kg group, at 8 weeks of treatment, rifampicin resistant strains were isolated from spleen of one out of four mice showing MIC at 2 mg/L. At 10 and 12 weeks, resistant strains were found from lung or spleen of two out of four mice showing MIC at 4–8 mg/L. These data were excluded from **Figure 4**.

To investigate the effects of increased dosage of rifampicin (10–50 mg/kg) on the post-treatment levels of persisters through RPF-induced resuscitation, we incubated lung and spleen homogenies at 6 and 12 weeks of antibiotic treatment with the culture filtrates. At 6 weeks post-treatment, only 50 mg/kg of rifampicin rendered lung CFU counts to zero, but 0.56 logs of RPF-resuscitable bacilli remained (**Table 2**). At 12 weeks post-treatment, incremental doses of rifampicin at 20, 30, and 50 mg/kg resulted in more effective elimination of RPF-dependent bacilli (1.33, 0.96, and <0.01 log cells/lung, 1.27, 1.09, and 0.5 log cells/spleen, respectively) after CFU counts in these organs were rendered negative (**Table 2**). Importantly, pulmonary sterility was observed after 12 weeks of high-dose rifampicin (50 mg/kg) treatment.

**Table 2:** Resuscitation of *M. tuberculosis* H37Rv in mouse lungs and spleens after treatment with different concentration of rifampicin.

		Lung		Spleen	
Treatment (mg/kg)	Weeks^∗^	Plate counts (log CFU/lung)	Broth counts RPF^†^ (log cells/lung)	Plate counts (log CFU/spleen)	Broth counts RPF^‡^ (log cells/spleen)
10	6	5.75 ± 0.11	7.49 ± 0.51	5.63 ± 0.29	7.36 ± 0.20
15	6	4.55 ± 0.14	5.97 ± 0.12	4.06 ± 0.13	5.28 ± 0.07
20	6	3.93 ± 0.09	5.02 ± 0.04	3.46 ± 0.19	4.53 ± 0.60
30	6	3.11 ± 0.39	4.09 ± 0.20	3.06 ± 0.58	4.06 ± 0.24
50	6	0	0.56 ± 0.23	0.38 ± 1.06	0.99 ± 0.45
10	12	4.98 ± 0.04	6.92 ± 0.22	5.03 ± 0.03	6.58 ± 0.50
15	12	2.41 ± 0.39	3.89 ± 0.23	2.09 ± 0.10	3.40 ± 0.40
20	12	0	1.33 ± 0.04	0	1.27 ± 0.98
30	12	0	0.96 ± 0.40	0	1.09 ± 0.47
50	12	0	<0.01	0	0.50 ± 0.05

RPF, resuscitation promoting factors; MPN, most probable number; ^∗^Weeks of rifampicin treatment. ^†,‡^Determined by MPN of the diluted lung and spleen homogenies with the culture supernatants containing RPF; <0.01, no bacilli were present in all sets of dilution tubes. Mean of MPN from three mice.

### High-Dose of Rifampicin in the Cornell Model

**FIGURE 5:**
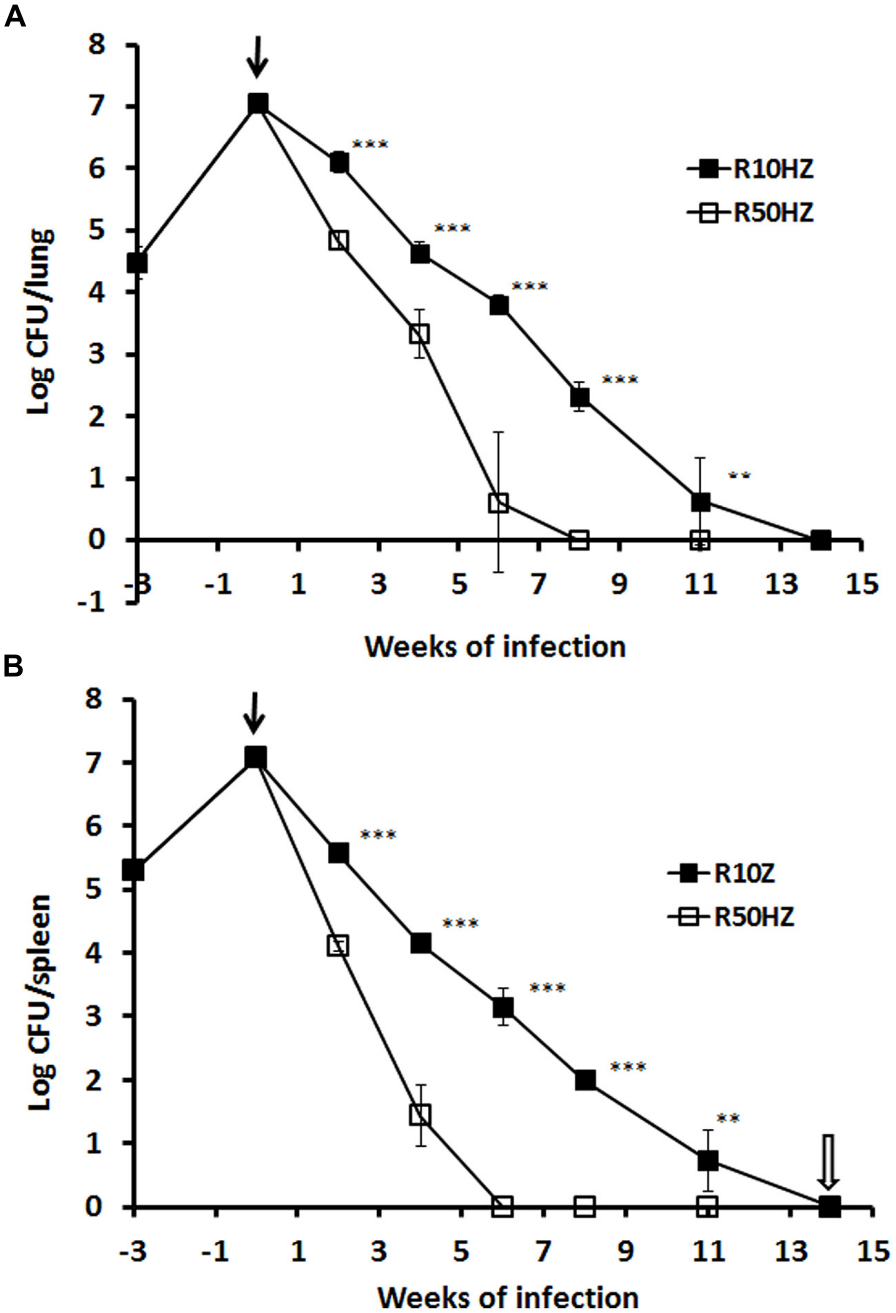
**Viability of *M. tuberculosis* H37Rv in the mouse Cornell model of dormancy.** The results of a single experiment are shown with viability expressed as log CFU counts per lung **(A)** and spleen **(B)**. Mice were infected intravenously at week –3 and the infection was allowed to progress for 3 weeks prior to treatment with isoniazid and pyrazinamide with rifampicin added at 10 or 50 mg/kg indicated as a solid arrow for 14 weeks (time weeks 0–14). At week 2, 4, 6, 8, 11, and 14 of post-treatment, CFU counts in the organs from each group were estimated. Steroid treatment was started immediately after the termination of 14 weeks of antibiotic treatment as indicated with an empty arrow. The experiment has been performed twice with reproducible results. Statistical analysis demonstrated that the decline of CFU counts in lungs and spleens was significant between low and high-dose rifampicin regiments, ^∗∗∗^*P* < 0.0001 or ^∗∗^*P* < 0.02 (Student’s *t*-test).

We compared the efficacy of high-dose (50 mg/kg) vs. low-dose (10 mg/kg) rifampicin in combination regimens with fixed standard doses of isoniazid (25 mg/kg) and pyrazinamide (150 mg/kg) in a well-defined tuberculosis model – the Cornell model. In terms of lung CFU counts, a 99% bacterial eradication (2-log reduction) was achieved more rapidly after treatment with high-dose (2 weeks) compared to low-dose (3.4 weeks) combination regimens. To achieve negative CFU counts in lungs, a longer period of treatment was required for the low-dose (14 weeks) compared to high-dose (8 weeks) regimen. This is shown in **Figure 5A**. In the sterile organs, no tubercle bacilli were recovered as confirmed by negative cultures of the organ homogenates in selective Kirchner broth for 4 weeks. Similar treatment profiles were seen in spleens (**Figure 5B**) except CFU count negativity was achieved slightly earlier in the high-dose treated group (6 weeks) compared to in lungs.

In order to investigate the effect of high-dose vs. low-dose regimens on the post-treatment level of persisters through RPF-induced resuscitation, lung homogenates at 8 and 14 weeks post-treatment were incubated with supernatants containing RPFs. At 8 weeks of treatment with the low-dose regimen, high levels of both bacterial CFU counts (2.32-logs in lungs; 1.99-logs in spleen) and RPF-resuscitated bacilli (3.88-log cells in lungs; 3.71-log cells in spleen) remained. At 14 weeks post-treatment, although CFU counts were negative, there were significant levels of RPF-resuscitated bacilli in lungs and spleens (1.41-log cells in lungs; 1.61-log cells in spleen). Treatment with high-dose regimen at both 8 and 14 weeks led to complete eradication of bacterial CFU counts. RPF-resuscitated bacilli (0.8-log) were only detectable in spleens at 8 weeks post-treatment. This effect is shown in **Table 3**.

**Table 3:** Resuscitation of *M. tuberculosis* H37Rv in mouse lungs and spleens of Cornell model after treatment with the 10 and 50 mg/kg rifampicin regimens.

		Lung		Spleen	
Treatment group	Weeks^∗^	Plate counts (log CFU/lung)	Broth counts RPF^†^ (log cells/lung)	Plate counts (log CFU/spleen)	Broth counts RPF^‡^ (log cells/spleen)
R10HZ	8	2.32 ± 0.24	3.88 ± 0.39	1.99 ± 0.07	3.71 ± 0.37
	14	0	1.41 ± 0.12	0	1.61 ± 0.29
R50HZ	8	0	<0.01	0	0.80 ± 0.36
	14	0	<0.01	0	<0.01

^∗^Weeks of treatment. ^†,‡^Determined by MPN of the diluted lung and spleen homogenies with the culture supernatants containing RPF; <0.01, no bacilli were present in all sets of dilution tubes. Mean of MPN from four mice.

### Relapse Rate after Treatment with the Regimens Containing Different Doses of Rifampicin in the Cornell Model

After 8 weeks of high dosage steroid immunosuppression which reactivated latent TB (McCune et al., 1966), disease relapse rates for the two dose rifampicin regimen treatments were determined by the percentage of mice that developed positive *M. tuberculosis* cultures in lungs, spleens or both. The organ CFU counts are shown in **Table 4**. The treatment with the regimen containing 10 mg/kg of rifampicin gave rise to *M. tuberculosis* positive organs in 21 out of 24 mice (87.5% relapse rate). In contrast, treatment with the regimen containing high-dose of rifampicin at 50 mg/kg resulted in zero counts in the organs showing relapse free (*P* < 0.001).

**Table 4:** Organ CFU counts after 8 weeks of steroid treatment.

Positive culture from	R10HZ	R50HZ
Spleen only	11	0
Lung only	5	0
Both organs	5	0
Neither organs^∗^	3	28
Total	24	28
Relapse (%)	87.5	0

R10HZ, Rifampicin 10 mg/kg, Isoniazid 25 mg/kg, Pyrazinamide 150 mg/kg for 14 weeks; R50HZ, Rifampicin 50 mg/kg, Isoniazid 25 mg/kg, Pyrazinamide 150 mg/kg for 14 weeks. ^∗^Plate and broth count negative.

## Discussion

A persister-eradicating drug regimen with a short duration of therapy would represent the most important advantage in tuberculosis treatment. To our knowledge, this study is the first to demonstrate, in the Cornell model, that combination therapy containing high-dose rifampicin (50 mg/kg) eradicates persistent bacilli, shortens treatment duration and reduces disease relapse. Furthermore, high-dose rifampicin treatment reduces the emergence of antibiotic resistance.

*In vitro, M. tuberculosis* persistence is driven by hypoxia (Wayne, 1994). In the *in vitro* stationary-phase model (Wayne, 1976, 1977; Hu et al., 2000), the bacilli grow initially in the top layer of an unagitated culture, where oxygen is available. They slowly adapt to microaerophilic and eventually to anaerobic conditions while sinking down in the medium and finally settling on the bottom of the containers (Hu et al., 1999). At 30–40 days, replication cannot be detected (Wayne, 1976, 1977). In late stationary-phase cultures after 60 days of incubation, protein synthesis is switched off (Hu et al., 1998). Interestingly, the data shown in the present paper indicates that the majority of bacilli entered into a viable but non-culturable stage (plate count negative), some of which was detected using 7H9 broth. However, a subpopulation of persistent *M. tuberculosis* was only resuscitated using the supernatant from actively growing cultures containing RPF (**Figure 1**). It has been shown that RPFs are bacterial self-generated stimulating proteins (Mukamolova et al., 1998) which not only facilitate the growth of young cultures but wake up dormant cells *in vitro* (Shleeva et al., 2002) and in TB patients’ sputa (Mukamolova et al., 2010). We conclude that the decline in CFU counts after 60 days of incubation (**Figure 1**) was not due to bacterial death, because the total viable cells (CFU counts and broth counts detected by RPF) remained constant (**Figure 1**).

In a murine infection model (Hu et al., 2006; Hu and Coates, 2009), it has been shown that the animal’s immune response restricts bacterial growth after 2–3 weeks of infection but fails to eliminate the infection driving the bacteria into an *in vivo* stationary phase. It has been reported that during chronic infection in mice, *M. tuberculosis* replicates and dies leading to a dynamic balanced growth (Gill et al., 2009). Our results showed that considerable amounts of bacilli, at least 15-folds more than CFU count positive bacteria, converted into RPF dependent and plate count negative persisters (**Figure 3**). 7H9 medium only resuscitated fivefolds of the plate count negative persisters. Our data confirmed that traditional viable counting methods greatly underestimate the actual numbers of viable cells. We previously demonstrated, by mRNA detection using reverse transcriptase polymerase chain reaction and [^3^H]uridine incorporation, that ongoing transcription continues in persistent *M. tuberculosis* in the sterile organs of the Cornell model (Hu et al., 2000). These results indicate that the persistent bacilli were metabolically active. It is not known if the persistent organisms we detected previously were the same as the RPF dependent persisters. These persistent bacteria contribute a major problem to the development of effective and shortened chemotherapy.

*In vitro*, stationary-phase cultures are profoundly tolerant to rifampicin. A 16-folds higher concentration of rifampicin (32 mg/L) was required to eradicate CFU count positive organisms compared to log phase bacteria (2 mg/L; **Figure 2**). Importantly, RPF dependent persisters in 100 days stationary-phase cultures could not be eliminated with even ultra-high concentrations of rifampicin (100 mg/L; **Figure 2C**). In mice, while increased doses of rifampicin alone after prolonged treatment were able to achieve organ CFU count negativity and reduced RPF-dependent bacteria; only high-dose of rifampicin (50 mg/kg) was able to eradicate the persisters in lungs (**Table 3**). Furthermore, we have shown here that in the Cornell model, high-dose rifampicin regimen eliminates all persisters, even the RPF dependent subpopulation (**Table 4**).

It has been found that in human tuberculosis prior to treatment, smear-positive sputa contained a considerable amount of RPF dependent persisters (Mukamolova et al., 2010). Furthermore, the numbers of RPF-dependent persisters was enhanced after standard chemotherapy compared with the plate counts positive populations (Mukamolova et al., 2010). It is not known whether the persisters we found *in vitro* and *in vivo* were the same as those in humans and if rifampicin treatment in mice necessarily predicts the situation with rifampicin in humans. We have recently highlighted (Coates et al., 2013) contradictory results with high-dosage rifamycin in mice and humans. Tuberculosis infections are different between mice and man, not least the absence of caseating granulomas in mice. Therefore, the interpretation of our data and the subsequent translation into human studies need to be undertaken with caution.

It has been proposed recently (Mitchison, 2012) that peak drug concentrations are better indicators than AUC for predicting higher degrees of persister-eradication and lower relapse rates. This is based on TB patients (rapid or slow acetylators) treated with isoniazid in whom the magnitude of clinical response was related to peak concentration of the drug (Mitchison, 2012). Repeated peaks can kill low-level resistant mutants. The same may be true for rifampicin (Mitchison, 2012) because repeated peak concentrations of rifamycins kill low antibiotic-tolerant persisters (Hu et al., 2000; Mitchison, 2012). Our results show that the PK value at 10 mg/kg gave a Cmax in blood of 11 mg/L and an AUC_0-24__h_ of 121 mg h/L which was very similar to those of previous studies in mice (Rosenthal et al., 2012; de Steenwinkel et al., 2013) and in humans (Doble et al., 1988; Sirgel et al., 2005). The PK values of rifampicin increased proportionally with increasing doses of rifampicin which was also in agreement with previous murine study (Rosenthal et al., 2012; de Steenwinkel et al., 2013). Although standard doses of rifampicin achieved a blood level of 11 mg/L, only 3.3% of free drug (de Steenwinkel et al., 2013) was able to diffuse to the lesion and inhibit bacterial growth as 97–98% of rifampicin or rifapentine was bound to plasma protein (Mitchison, 1998; de Steenwinkel et al., 2013). This suggests that a standard dose of rifampicin is not high enough to kill persistent organisms which are present at the beginning of the treatment and formed during the antibiotic treatment. In the murine model, we showed here that increasing dose of rifampicin exhibited an accelerated dose-dependent eradication of persistent bacteria (**Table 3**). When the rifampicin concentration was increased to 30 mg/kg or higher, high blood Cmax was achieved, leading to a higher level of biological available rifampicin which was able to remove persistent bacteria (Mitchison, 2012).

We showed rifampicin resistance appeared after 8 weeks of standard rifampicin treatment (10 mg/kg) in mice which was exacerbated by increasing the duration of therapy. This concurs with previous evidence that prolonged anti-TB drug exposure and higher bacterial burdens predict the emergence of antibiotic resistance (de Steenwinkel et al., 2010). In certain countries, standard anti-TB drug regimens rendered a low 2-months sputum culture conversion rate (between 50 and 70%) which led to acquired drug resistance (Abdool Karim et al., 2010; Calver et al., 2010). The development of rifampicin resistance was strongly associated with lower serum drug concentrations (Pasipanodya et al., 2013), while higher Cmax and AUC inhibited rifampicin resistance (Gumbo et al., 2007). Importantly, we show here that higher doses of rifampicin (>15 mg/kg) induced rapid bacterial eradication with undetectable resistant bacilli. Increasing the dose of rifampicin significantly reduced the risk of antibiotic resistance emergence. The precise reason for this mechanism is unclear; we speculate it relates to the fact that dead, and possibly, metabolically inhibited bacteria are unable to mutate.

We present unprecedented evidence that true TB-sterility, i.e., negative CFU count and RPF-resuscitatable cultures, can be achieved in lungs and spleens after 8 and 14 weeks of combination chemotherapy containing high-dose rifampicin (50 mg/kg). For the first time, we offer a possible mechanistic insight into this observation. This builds significantly on existing evidence that suggests that high-dose rifampicin shortens treatment duration from 14 to 8 weeks (Rosenthal et al., 2012; de Steenwinkel et al., 2013). Our findings show that RPF-dependent bacilli constitute a major pool for disease relapse in the mouse Cornell model, not only the culturable but also the non-culturable bacilli are eradicated, the treatment period is shortened and disease relapse rate is reduced.

## Conclusion

We demonstrated that RPF-dependent persistent *M. tuberculosis* was formed *in vitro* and *in vivo*. The current recommended dosage of 10 mg/kg is insufficient to kill persistent bacilli. Higher doses of rifampicin alone or in combination with isoniazid and pyrazinamide significantly shortened the treatment period and eliminated disease relapse by removing persistent bacteria. Optimizing rifampicin to its maximal therapeutic efficacy with acceptable side-effect profiles will provide valuable information in human studies and can potentially improve current tuberculosis chemotherapy.

## Conflict of Interest Statement

The authors declare that the research was conducted in the absence of any commercial or financial relationships that could be construed as a potential conflict of interest.
